# Functionalized biochar from waste as a slow-release nutrient source: Application on tomato plants

**DOI:** 10.1016/j.heliyon.2024.e29455

**Published:** 2024-04-09

**Authors:** Domenico Rosa, Valerio Petruccelli, Maria Cristina Iacobbi, Elisa Brasili, Camilla Badiali, Gabriella Pasqua, Luca Di Palma

**Affiliations:** a Department of Chemical Engineering Materials Environment & UdR INSTM, Sapienza-Università di Roma, Via Eudossiana 18, 00184, Roma, Italy; b Department of Environmental Biology, Sapienza-Università di Roma, Piazzale Aldo Moro 5, 00185, Roma, Italy

**Keywords:** Functionalized biochar, Slow-release fertilizers, Desorption, Plant nutrition, Circular economy, *Solanum lycopersicum*

## Abstract

Licorice processing waste was pyrolyzed at different temperatures (500 and 700 °C) to obtain biochar (BC500 and BC700) for use as a slow-release fertilizer on *Solanum lycopersicum*. The materials were characterized through BET analysis, SEM, elemental analysis, pH_zc_, and pyrolysis temperature effect was evaluated. The biochars were functionalized by the impregnation method to enrich them with nitrogen, phosphorus, and potassium (NPK), and desorption tests were performed in aqueous solution at different pHs (5 and 7). The pseudo-second-order model described well the release of all 3 macronutrients tested, BC500 was found to have slower release kinetics due to smaller pore size, reaching adsorption/desorption equilibrium after 14 days, compared with 10 for BC700, K_des_ were lower in all 3 cases and NPK content was higher, initial pH did not change the release kinetics. BC500 was selected as an agricultural soil conditioner by testing at both different dosages of BC (0–25 %) and different NPK ratios (3:1:4 and 4:1:3). The treatment significance was evaluated. The best treatment resulted in BC dosage of 25 % nutrient ratio 4:1:3 which increased, compared to the control, total chlorophyll content (+38 %) and carotenoids (+15 %).

## Highlights

• Biochar properties (pH_ZC_, surface area, and nutrient content) strongly depended on the pyrolysis temperature.
• Biochar synthesized at 500 °C showed the lowest desorption constant (K_des_).
• K, N, and P release by biochar followed a second-order kinetic.
• Functionalized biochar at 500 °C (NPK ratio 4:1:3) increased total chlorophyll content by 38 %.
• Functionalized biochar at 500 °C (NPK ratio 4:1:3) increased carotenoids content of 15 %.

## 1 Introduction

The increase in world population over the past fifty years has led to negative consequences for the environment and agricultural production as an arable land reduction, which has necessitated the adoption of intensive agricultural practices to improve production efficiency [[1], [2], [3]]. However, traditional chemical fertilizers overuse causes environmental problems, such as water pollution by nitrogen (N) and phosphorus (P) fertilizers due to their high solubility in water [4], resulting in water eutrophication (disproportionate microalgae growth in suspension) [5] because plants are unable to absorb all the macronutrients provided immediately [6]. Furthermore, conventional fertilizers contribute to the accumulation of unused macronutrients in soil leading to potential repercussions for human health. Recent observations of high nitrate amounts in soil (especially in agricultural areas) have raised concerns because their conversion into nitrites can be problematic. Nitrites can bind to hemoglobin, a blood protein that transports oxygen throughout the body, transforming it into methemoglobin, which is unable to transport oxygen. Furthermore, nitrates can turn into nitrosamines, substances that pose a possible carcinogenic risk [7].

**Fig. 1:**
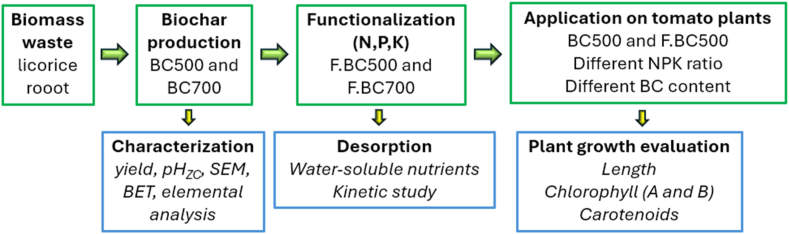
Research summary flowchart.

Slow-release fertilizers offer a promising solution to environmental issues associated with conventional fertilizers. By continuously supplying nutrients to plants, they diminish macronutrient leaching, mitigating water pollution and microalgae proliferation [8,9]. Biochar, due to its porous structure and large surface area, is an excellent nutrient carrier such as nitrogen, phosphorus, and potassium [10]. As a result, biochar in agriculture has gained increasing popularity over the past two decades [[11], [12], [13]]. Biochar, a porous carbon-rich material produced through the pyrolysis of various carbon-based raw materials [14] including food processing waste, animal manure [15], or crop residues, enhances agronomic properties and soil fertility [16], thereby boosting crop productivity [17]. Its unique carbon structures, notably a higher proportion of aromatic carbon and condensed aromatic structures compared to other organic matter like lignin, contribute to its stability [18]. Various forms of condensed aromatic structures, including amorphous carbon, turbostratic carbon (formed at higher temperatures), and graphite carbon [19], further enhance its versatility. Biochar characteristics such as high biodegradability, high total and organic carbon content, optimal micro and macroelement concentrations, large specific surface area, surface functional groups, pH, and porosity [20], enable its role in the circular economy concept. By being derived from waste, biochar facilitates proper waste management and valorization while also aiding carbon sequestration, reducing greenhouse gas emissions, and mitigating climate change [21]. In addition to providing carbon, biochar plays a crucial role in plant growth by supplying essential macronutrients [22,23]. Enhanced nutrient uptake, particularly nitrogen (N), phosphorus (P), and potassium (K), in soils amended with biochar results from increased nutrient availability and microbial activity in the nutrient cycle, impacting enzymatic activity [24]. Large pores originating from the vascular bundles of raw biomass improve soil quality and provide habitats for symbiotic microorganisms [20]. Furthermore, biochar adsorption capacity allows for the regulation of nutrient quantity and ratio, making it a highly versatile material. Distinguishing between woody and non-woody biomass is crucial in biochar production [20]. Woody biomass, including forestry residues and tree materials, features low moisture and ash content, high bulk density, and fewer pores [25]. Non-woody biomass, consisting of crops and agricultural residues, animal waste, and municipal and industrial solid waste, typically has higher moisture and ash content, richer macro and micro-nutrient content, lower calorific value, lower bulk density, and more pores. Lower moisture content is advisable for biochar production due to reduced thermal energy and time required for pyrolysis, making the process economically viable [26]. Moisture can also affect structural properties; for example, with decreasing moisture in maple bark, the carbon surface becomes more polyaromatic and graphite-like, likely due to the longer effective pyrolysis time after water evaporation. Therefore, considering these factors, biochar derived from non-woody biomass is recommended for agricultural purposes due to its inherently higher nutrient content. The biochar used in soil improves fertility status, quality, and water retention capacity [27] owing to its porous structure ability to retain water. It influences various soil properties [28] including pH, EC (Electrical Conductivity), CEC (Cation Exchange Capacity), O:C ratio, NPK, soil organic matter, and soil biological activity [29]. Soil pH increases after applying biochar due to the activity of negatively charged phenolics, carboxyl, and hydroxyl groups on biochar surfaces, which bind H^+^ ions in the soil [30,31]. However, excessively high soil pH could decrease phosphorus, magnesium, and molybdenum bioavailability. Biochar produced with slow pyrolysis (400–600 °C) positively influences soil aggregation in various soils [[32], [33], [34]]. Although biochar offers numerous benefits, its nutrient composition may not always meet plant requirements, necessitating techniques like functionalization to enhance its efficiency in agriculture [35]. However, is necessary to highlight some feedstocks known for their high concentration of specific nutrients [36]. For example, Zwetsloot et al. (2016) focused on producing biochar rich in phosphorus (P) from bone waste, achieving P contents ranging from 13 % at 350 °C to 15 % at 750 °C [37]. Ma and Matsunaka (2013) obtained biochar with a total P content of 10 % from dairy cattle carcasses (a skin, meat, and bone mixture) processed at 450 °C [38]. *P*-enriched biochar was also produced through the pyrolysis of bacterial biomass waste from *Escherichia coli*, resulting in a P concentration of 84.7 mg/g, approximately 11 times higher than the original biomass [39] due to the use of K_2_HPO_4_ and KH_2_PO_4_ in the culture medium and P released from the biomass cells decomposition. Karim et al. (2017) investigated potassium (K) enrichment in biochars produced by thermal processing of banana peduncle biomass under different gases (oxygen and argon) and processing times (up to 9 min) [40]. The available K content in banana peduncle (66 mg/g) increased to 86, 164, and 259 mg/g in biochar produced by argon plasma processing with residence times of 3, 5, and 7 min, respectively. Additionally, Mosa et al. (2018) produced functionalized biochars with high phosphate recovery potential from *Eichhornia crassipes* grown in synthetic contaminated water. Physicochemical analyses confirmed in-situ functionalization, resulting in increased specific surface area, positive functional groups, and anion exchange capacity (AEC). Consequently, the functionalized biochar exhibited enhanced nutrient supply compared to the unfunctionalized forms [41]. Recent studies have demonstrated nutrient-loaded biochar can behave as a slow-release fertilizer. For instance, Yao et al. showed that biochar from sawdust functionalized with nitrogen and phosphorus can act as a slow-release fertilizer [42]. Zhang et al. also functionalized biochar produced from waste derived from the distilled spirits industry with nutrients such as nitrogen, phosphorus, and potassium in a ratio of 5:3:7 testing biochar in eggplant cultivation [35]. In many cases, not all mechanisms are clear and there are many aspects to be investigated; this is difficult given the wide variability of crops and their different requirements, as well as the wide variability of biochar. Therefore, further studies are appropriate to clarify these aspects, such as the optimal biochar dosage, the optimal nutrient ratio, and especially how biochar properties can be engineered according to the treatment and different nature of the biomass to optimize the use of biochar for crops,

Given the great biochar potential the present study aimed to synthesize simply and economically a slow-release biochar-based fertilizer from licorice processing waste embracing the circular economy concept and verifying its effect on tomato plant growth. Investigation of the characteristics of the biochar produced at different pyrolysis temperatures and the development of a simple and cost-effective functionalization procedure were preliminarily performed. A material chemical and physical characterization and evaluation of nutrient release mechanisms and kinetics through batch tests were carried out on both biochars. Finally, an assessment of plant morphological and physiological parameters throughout the experimental period is illustrated in Fig. 1.

Taken into account of this study to quantitatively evaluate the outcomes of the research were the easiness and reproducibility of the synthesis and functionalization method, the rate of desorption nutrients, and the enhancement in fitness (chlorophyll and carotenoids) of plant growth.

## 2 Materials and method

### 2.1 Biochar synthesis

**Fig. 2:**
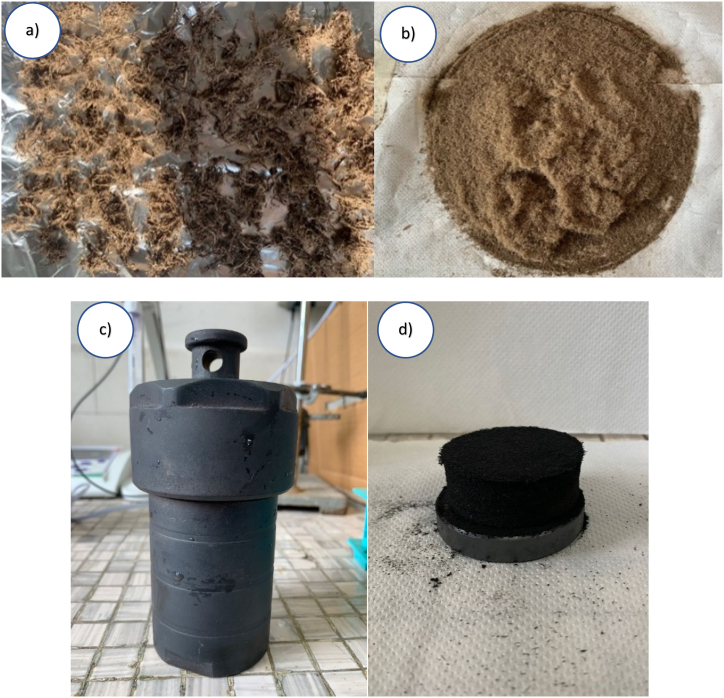
**a)** Raw licorice roots; **b)** roots ground and sieved with a 1.19 mm sieve; **c)** autoclave used for licorice pyrolyzation to obtain anoxic environment; **d)** biochar obtained after 1 h of pyrolyzation (at 500 and 700 °C).

Biochar using licorice root waste was produced (Fig. 2a). Before the pyrolisis, the roots were thoroughly washed with water to remove any impurities, with the final wash being done with demineralized water. The washed roots were then dried overnight at 60 °C and ground into a fine powder using a coffee grinder. The resulting powder was passed through a 1.19 mm mesh sieve (Giuliani Tecnologie) to obtain uniform size (Fig. 2b) and compacted into an autoclave (Fig. 2c) to ensure an anoxic environment and subjected to pyrolysis in a muffle furnace (slow pyrolisis) for 1 h at two different temperatures: 500 °C and 700 °C (Fig. 2d). This process resulted in two biochar types, BC500, and BC700, respectively, and have been ground in a mortar to obtain a homogeneous powder.

### 2.2 Biochar characterization

Scanning Electron Microscopy (SEM) analysis was conducted on both biochars (BC500 and BC700) using a Zeiss Auriga instrument (Zeiss, Oberkochen, Germany). The sample surface was pre-coated with ∼20 nm of gold using a Quorum Q150T ES (Quorum Technologies, Ltd., Laughton, East Sussex, UK) and subsequently examined at an acceleration voltage of ∼2 keV.

Brunauner Emmett–Teller (BET) surface areas (S_BET_) were obtained from N_2_ adsorption at 77 K using Quantachrome Autosorb-1.

The amount of carbon in samples pyrolyzed at different temperatures was quantified using an Elemental Analyzer Euro EA.

To evaluate N, P, and K content acid leaching was performed on the ash obtained from burning at 500 °C for 1 h of 1.00 g of BC500 and BC700 in 100 mL of 1.2 M hydrochloric acid at 60 °C for 2 h. The sample was filtered with 2–3 μm cellulose filter paper and the liquid stream was analyzed to quantify N, P, and K concentration using Induced Coupled Plasma (ICP) PerkinElmer Avio 220.

The zero-charge pH (pH_zc_) was determined for samples BC500 and BC700 as in previous work [43] by placing 0.1 g of BC in contact with 10 mL of solutions at different pH values based on demineralized water for 5 days. The solutions by acidification with HCl and alkalinization with NaOH were prepared. Solution pH was measured at the end of the predetermined time using a benchtop pH meter (Crison GLP 421) and a final pH versus initial pH graph was constructed.

### 2.3 Biochar functionalization

BC500 and BC700 using the impregnation method were functionalized [42], whereby 18.3 g of biochar was impregnated with a mixture of 0.325 g of potassium dihydrogen phosphate (KH_2_PO_4_), 0.353 g of potassium hydroxide (KOH), 0.435 mL of nitric acid (HNO_3_ 65 %), and 0.383 g of ammonium nitrate (NH_4_NO_3_). The dosages of each substance were adjusted to obtain an NPK nutrient weight ratio of 3:1:4 (BCA) and 4:1:3 (BCB, for which 0.325 g of potassium dihydrogen phosphate, 0.185 g of potassium hydroxide, 0.227 mL of nitric acid and 0.714 g of ammonium nitrate were used). The reagents were dissolved in 18.3 mL of deionized water and poured over the biochar, and the mixture was left to soak for three days, then the biochar was dried in an oven at 60 °C overnight.

### 2.4 Kinetic desorption experimental set-up

To evaluate the desorption kinetics of water-soluble nutrients (NH_4_^+^, K^+^, NO_3_^−^, PO_4_^3−^) and their availability over time, batch tests were carried out placing 0.50 g of both functionalized (F.BC with nutrient ratio NPK 3:1:4) and unfunctionalized (BC) pyrolyzed at different temperatures (500 and 700 °C) in a sealed container with 50 mL of demineralized water at different pH (5–7) and left in contact for a predetermined time (1–35 days) without stirring. After, the samples were filtered with 2–3 μm cellulose filter paper, and the liquid stream, containing the nutrients, was analyzed by atomic absorption spectrometry (AAS) for K^+^ evaluation and ion chromatography (IC, Thermo Fisher Scientific) for nitrate and phosphate ion evaluation (NO_3_^−^ and PO_4_^3−^, respectively). Ammonium ion content (NH_4_^+^) was evaluated by a spectrophotometric method using Nessler's reagent made as in previous work [44] by the following procedure.

• 3.5 g KI in 10 mL of demineralized water
• 1.4 g HgCl_2_ in 35 mL of demineralized water
• 12.0 g of KOH in 25 mL of demineralized water
• Demineralized water until 100 mL

Nessler's reagent in a ratio of 1 mL of reagent to 50 mL of solution was added. After 10 min, the time required to get the reaction done the sample has changed its color depending on the concentration of NH_4_^+^.

pH measurements were also carried out on the solutions at different times.

### 2.5 Kinetic study

The kinetics of nutrients (K, N, and P) desorption study from nutrient-loaded biochars (F.BC500 and F.BC700) was carried out using an empirical second-order kinetics model to fit and accurately represent the nutrient concentration over time data. Therefore, the following equation was employed (Equation (1)):

(1) $$\frac{dC_{t}}{dt}=K_{des}{\left(C_{e}-C_{t}\right)}^{2}$$

where K_des_ represents the second-order rate constant (L·mg^−1^·days^−1^), C_t_ is the concentration at a specific time and C_e_ is the equilibrium concentration (mg·L^−1^). Subsequently, Equation (1) was solved by integrating from t = 0 to t = t and from C_t_ = 0 to C_t_ = C_t_, and by rearranging the terms, Equation (2) was obtained.

(2) $$C_{t}=\frac{{C_{e}}^{2}K_{des}t}{C_{e}K_{des}t+1}$$

where t is the elapsed time (days). C_e_ and K_des_ values were obtained by minimizing the sum of squared differences between the observed concentration and the predicted concentration from the model [45].

### 2.6 Fertilization experimental set-up

The experiment was performed under laboratory conditions using tomato seeds from *Solanum lycopersicum* cultivar Ciliegia purchased from the Blumen S.P.A (Milano, Italy). Firstly, tomato seeds into Petri dishes with a sterilized filter paper double layer were transferred. The seeds were kept in continuous darkness at a constant temperature of 25 °C for 72 h. After germination, the seedlings were maintained in Petri dishes for 96 h under a 16/8 photoperiod (approximately 130 μmol m^−2^ s^−1^). Subsequently, tomato seedlings (n = 5 for each treatment) were transferred in black pots (34 cm × 23 cm) containing the soil (COMPO SANA®, Germany) amended with functionalized (F.BC500) and unfunctionalized (BC500) biochar at rate of 5 %, 15 % and 25 % (w/w). The experiment included four treatments namely: control (without any amendments), unfunctionalized biochar BC500 (BC0), functionalized biochar (F.BC500) type A (BCA) and functionalized biochar (F.BC500) type B (BCB). The plantlets were irrigated with tap water until the end of the experiment (28 days) and chlorophyll and carotenoids content was evaluated. Plant height was determined every 7 days by the ImageJ software as described by Schneider et al. [46]. To assess the carotenoid and chlorophyll content in the leaves, the samples were weighed and added to 96 % (v/v) methanol at a ratio of 1:50. The samples were kept in the dark at 4 °C for 72 h, and then the supernatant was collected and analyzed with a Shimadzu UV-1280 spectrophotometer. Chlorophylls *a* and *b* were measured at 653 nm and 666 nm, respectively, while total carotenoids were measured at 470 nm [47]. Quantification was performed according to the formulas of Lichtentaler and Wellburn was performed [48] (Equations 3-5).

(3) $$Chlorophyll\;A:Ca=15.65∙A_{666nm}-7.34∙A_{653nm}$$

(4) $$Chlorophyll\;B:Cb=27.05∙A_{653nm}-11.21∙A_{666nm}$$

(5) $$Total\;carotenoids:Cc=\frac{1000∙A_{470nm\;}-2.86∙Ca-129.2∙Cb}{245}$$

### 2.7 Quantification and statistical analysis

The significance of the difference among the treatments was tested by analysis of variance (two-way-ANOVA). Normality and Holm-Sidak multiple range tests at p < 0.05 were performed using Sigma Plot (version 12.0, Sysstat Software, Inc., USA).

## 3 Results and discussion

### 3.1 Biochar characterization: SEM, yield, composition, and BET

**Fig. 3:**
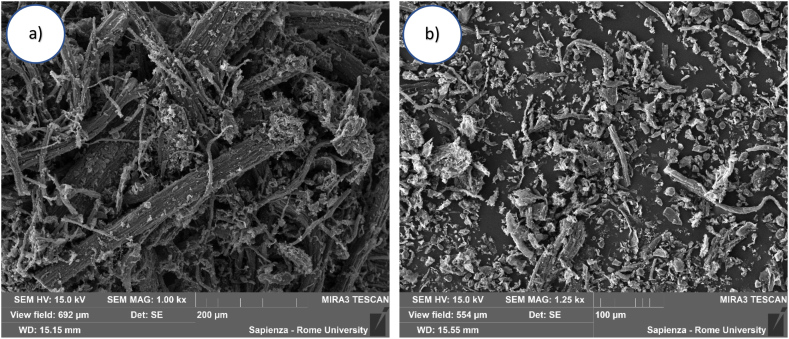
SEM microrography of **a)** BC500 and **b)** BC700 samples.

The biochar was characterized using SEM (Fig. 3), resulting in particles highly heterogeneous, exhibiting irregular shapes and significant geometric variability [49]. They range from long particles up to 400 μm to grains of about 10 μm, with a filamentous morphology that reflects the starting matrix (Fig. 3a). However, this morphology tends to be less evident for the sample pyrolyzed at 700 °C (Fig. 3b), as the matrix has undergone a more drastic modification due to the temperature effect.

The surface for both samples appeared rough and not very smooth, with a slightly pronounced porosity. Nevertheless, the smaller grain size of BC700 would justify a larger surface area.

The biochar production method used in the study was produced, compared to the initial biomass, 33 % of BC500 and 27 % of BC700. An inverse relationship between biochar yield and pyrolysis temperature was observed [50]. As the pyrolysis temperature increased, the quantity of biochar produced decreased [51,52]. This is primarily due to the loss of volatile components such as CO, H_2_, and CH_4_ [53] mainly based on H and O. This would explain the increase in carbon content observed in the samples as the pyrolysis temperature rises and the progressive decrease in H. The sample at 500 °C had a C content of 69 % and an H content of 3 %, while the biochar pyrolyzed at 700 °C had a C content of 74 % and an H content of 1 %.

Acid digestion tests of the BC samples resulted in the nutrient content shown in Table 1.

**Table 1:** Total amount of nutrients in BC500 and BC700 based on ICP analysis after ashes acidic mineralization (ashes from 1.00 g of BC in 100 mL of 1.2 M hydrochloric acid at 60 °C for 2 h).

Sample	[mg/g]			
	K	N	P	Fe
**BC500**	10.3 ± 1.0	4.9 ± 0.3	4.6 ± 0.4	3.7 ± 0.6
**BC700**	7.7 ± 0.5	4.2 ± 0.2	1.1 ± 0.3	4.8 ± 0.5

From Table 1, a slight difference in nitrogen content between BC500 and BC700 samples can be observed, increasing pyrolysis temperature, where the nitrogen content decreased from 4.9 to 4.2 mg/g because nitrogen volatilization begins at around 200 °C [52,54] as it is associated with organic molecules decomposing due to temperature effects. The release of nitrogen from the carbonaceous matrix, primarily in the ammonium form [54] seems to be appreciable even at low pyrolysis temperatures (270 °C) [55]. Therefore, at 500 °C, it is plausible the biochar may have already lost nitrogen in the form of more volatile and unstable compounds [50] with most nitrogen present in the form of aromatic heterocycles and thus less volatile [56]. However, the temperature increase still contributes partially to nitrogen volatilization. In a related study, Lang et al. (2005) observed a reduction in nitrogen content by half from around 400 to 750 °C due to volatilization [55]. It is also worth considering this effect is tempered by the loss of other elements such as oxygen and hydrogen, as well as the overall decrease in biochar production yield at high temperatures, which tends to concentrate heteroatoms present in the biochar. On the contrary, potassium (K) [52] and phosphorus (P) volatilization occurs at 700 °C [54]: consequently, since at 500 °C most of the phosphorus and potassium initially present in the matrix were not volatilized, only at 700 °C a significant reduction of P and K content was observed.

Iron, although not relevant to this study, was analyzed as a non-volatile component to confirm the inorganic constituents amount remaining in the biochars increased with increasing temperature [54]. As the biomass concentration increases (from 3.7 to 4.8 mg/g), by the decrease in mass yield, the biochar loses oxygen and hydrogen to enrich the non-volatile inorganic fraction [52].

The BET analysis carried out on the samples pyrolyzed at different temperatures revealed an increase in specific surface area with increasing pyrolysis temperature (Table 2). This increase can be due to the decomposition of lignocellulosic materials, particularly hemicellulose, and cellulose, which are mainly converted into gaseous products during the pyrolysis process, while lignin is primarily converted into carbon [54]. Additionally, hydrogen (H), oxygen (O), and inorganic mineral substances evaporation contribute to an increase in specific surface area and pores formation resulting in a porous structure in biochar, as reported by several authors [[57], [58], [59]]. However, the specific surface area increase is highly dependent on the starting material. Indeed, it is true that up to temperatures of approximately 800 °C, there is an increase in specific surface area. However, beyond this temperature range, a collapse and sintering of the biochar structure occur, resulting in a decrease in surface area. This phenomenon is commonly observed and is due to the thermal degradation and restructuring of the biochar at higher temperatures [[60], [61], [62]].

**Table 2:** BET analysis, surface area, pore volume, and pore size of biochars produced at different temperatures.

Sample	BET surface area (m^2^/g)	Pore volume (cm^3^/g)	Pore size (nm)
**BC500**	13.1	0.011	7.8
**BC700**	21.4	0.052	30.2

### 3.2 pH and zero charge pH results

**Fig. 4:**
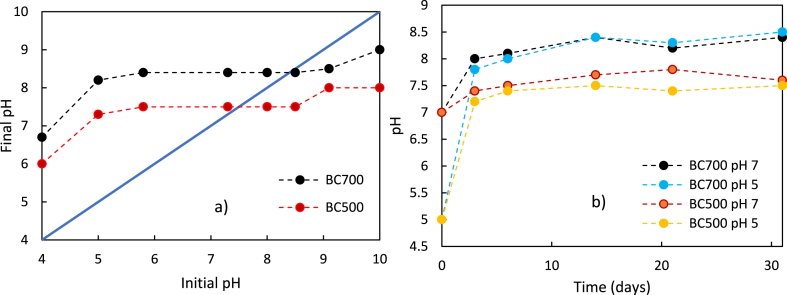
**a)** Initial pH vs final pH made by measuring the pH of a 10-mL solution in contact with 0.1 g BC for 5 days. **b)** pH variation of the solution over time, starting from different pH values using 0.1 g of BC500 and BC700 in 10 mL of solution.Figu significant amount of the residual nitrogen in the biochar was likely present as heterocyclic aromatic structures [8] in which nitrogen can have a non-bonding electron pair, which can act as an electron donor site during chemical reactions or be involved in bonds with acids or other electron acceptors, behaving as a Lewis base. Consequently, N-containing heteroaromatic rings can exhibit basicity characteristics, interacting with acids or electron acceptors through the donation of the non-bonding electron pair [14]. In addition, the higher temperature during the carbonization process may have reduced acid functional groups on the biochar surface. In turn, these acidic groups were predominantly replaced by basic groups, such as quinone [50], thus contributing to an increase in the pH_zc_. Another factor contributing to biochar alkalization is carbonate production [51] promoted by high temperature and the increase in alkali non-volatile metals such as Ca, Mg, and Na [63] in biochar.

The results of the tests performed at different pH to determine the zero-charge pH are shown in Fig. 4.

Fig. 4b demonstrates the solution pH tends to converge towards the pH_zc_, regardless of the initial pH (pH 5 and pH 7 were tested). This finding demonstrates that biochar can act as a buffering agent and pH regulator [64] due also to organic anions, specifically -COO^–^ and -O^–^ groups, on the surface of biochar [51]. It is an interesting result as it indicates that biochar can be engineered and modulated to achieve the desired pH in soil, thereby meeting the specific pH requirements of different plant crops and microbial cultures [14]. Moreover, this pH modulation can be achieved through variations in the pyrolysis temperature, adding a level of control and customization to biochar production [50].

### 3.3 Desorption and kinetic study

Tests to assess the desorption of available nutrients using non-functionalized biochar were carried out.

The performed experiments at different initial pH (5 and 7) did not show a significant influence on nutrient desorption due to biochar buffering properties within the investigated pH range. Therefore, it can be concluded the solution pH did not significantly affect the desorption kinetics.

The results shown in Table 3 demonstrate that, over a span of 35 days, the biochar desorbed nutrient quantities below the maximum theoretical content as previously determined in the preliminary acid digestion tests (Table 1). This difference can be due to the lower availability of water-soluble nutrients, as not all nutrients are present in soluble forms. A significant finding is a noticeable difference between BC500 and BC700, with BC700 exhibiting lower nutrient content, because, as earlier discussed, at 700 °C the recalcitrant aromatic heterocyclic structures production [56] in which nitrogen is less readily available than amine nitrogen was promoted. Consequently, high-temperature pyrolysis limits the availability of nitrogen that biochar is willing to release [8].

**Table 3:** Nutrients desorbed by 1.00 g of biochar in 100 mL of water over 35 days.

Sample	[mg/L]		
	K	N	P
**BC500**	60.6 ± 3.7	24.5 ± 1.3	21.9 ± 2.8
**BC700**	49.7 ± 5.2	14.0 ± 1.6	3.9 ± 0.4

The higher temperature also decreased phosphorus availability, probably due to its conversion into a less water-soluble material. Increasing temperature increases crystallized minerals formed to associate with P, creating less soluble P forms. For instance, the soluble salt KH_2_PO_4_ present up to 500 °C is converted to magnesium cyclo tetraphosphate (Mg_2_P_4_O_12_) at higher temperatures [52], known for its thermal and chemical stability and insolubility, but also Mg–P crystals in the forms of MgHPO_4_ and Mg(H_2_PO_4_)_2_ [65] or to Ca-associated minerals (Ca_3_(PO_4_)_2_). For potassium, the effect is less pronounced, and it is mainly due to the loss of available potassium caused by evaporation.

Experiments were performed to evaluate the desorption kinetics of functionalized biochar (F.BC): The desorbed nutrient percentage using the following equation (Equation (6)) was calculated:

(6) $$\%\;\text{release}=\frac{C\left(t\right)}{Cmax}\;x100$$

where C(t) represents the nutrient (K, N, or P) concentration at time t and C_max_ represents the concentration that would be obtained if all nutrients were desorbed. C_max_ was calculated as the sum of the added nutrients (the same for both BCs: 4.0 mg of P, 18.5 mg of K, and 12.1 mg of N per gram of BC added through the impregnation method) and the naturally present amount quantified through acid digestion tests (Table 1). The difference between BC500 and BC700 is due to nutrient depletion by BC700 resulting from the higher temperature treatment, therefore the C_max_ of BC700 of the 3 nutrients examined all resulted lower than the C_max_ of BC500.

Specifically, for F.BC500, the C_max_ are as follows: K - 288 mg/L, N - 170 mg/L, and P - 86 mg/L. For F.BC700, the C_max_ are: K - 262 mg/L, N - 163 mg/L, and P - 51 mg/L, considering 0.5 g of F.BC in 50 mL of water.

**Fig. 5:**
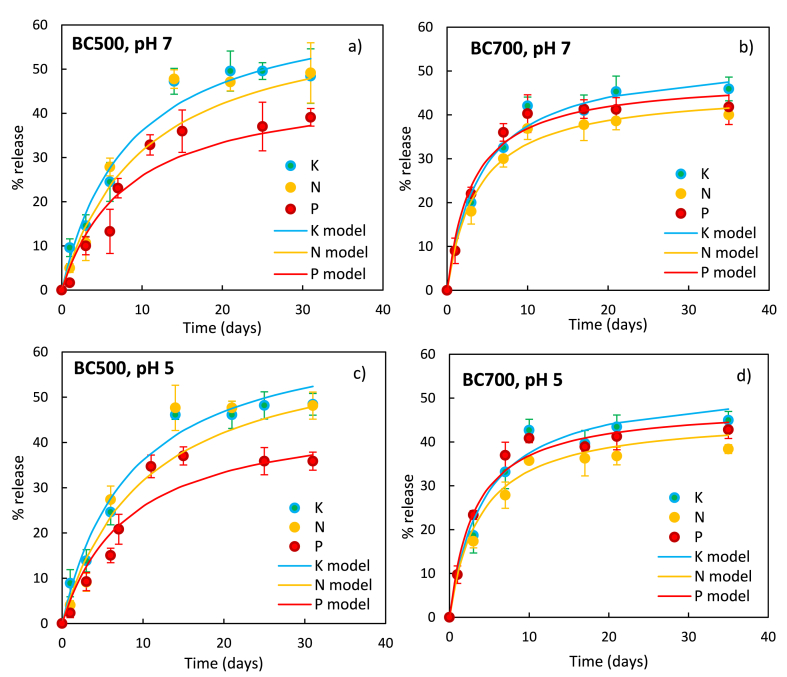
Desorption kinetics in different samples at different pH: a) F.BC500, pH 7; b) F.BC700, pH 7; c) F.BC500, pH 5; F.BC700, pH 5. The continuous curves represent the second-order kinetic model applied to fit the data.

Experiments carried out at different initial pH values (5 and 7), shown in Fig. 5, did not show a significant influence of pH on nutrient desorption, therefore, in subsequent discussions related to kinetics, only data obtained at pH 7 was processed.

The kinetic study revealed the N, P, and K desorption from functionalized biochars steadily increased over time, reaching an equilibrium at approximately 10 days for BC700 (Fig. 5b and d) and 14 days for BC500 (Fig. 5a and c) resulting in an effective controlled release behavior due to the porous structure of biochar that results in hindered diffusion of all nutrients used. This implies that due to strong different type interactions, biochar can retain nutrients and significantly reduce leaching losses [66]. For example, the controlled release of P can be due to the precipitation of the phosphate ion on Mg- and Ca-based clusters present in the biochar. The strong interaction with iron could also play an important role, which adsorbs phosphorus through Fe–OH bonds and forms complexes (Fe–*O*–P). Nitrogen release in ammonium form is controlled by hydrogen bond type interaction and electrostatic interactions [67].

No significant differences were observed in the desorption of the various nutrients, while the different biochars exhibited distinct behaviors. Specifically, BC500 achieved adsorption/desorption equilibrium over a longer period (approximately 14 days) compared to BC700 (10 days) [52,65]. This was due to the reduced pore size of BC500 compared to BC700, as observed in other literature studies [45,68] and in agreement with the results of the BET analysis (Table 2). In addition, the greater presence of oxygen-based functional groups in BC500 than in BC700 that interact with nutrients adsorbed on the surface by retaining and complexing them [67].

Furthermore, the nutrient desorbed, shown in Table 4, was lower in BC700, consistent with previous findings, indicating a reduced nutrient leachability (due to the formation of more stable and less soluble species due to temperature effect [8]) as well as a lower nutrient content.

**Table 4:** Percentage nutrient desorption: equilibrium concentration (C_e_) observed after 35 days relative to the theoretical maximum concentration.

Sample	% [C_e_/C_MAX_]		
	K	N	P
**BC500**	63.8	47.1	62.8
**BC700**	46.1	48.4	55.2

Leachability reduction was most pronounced for nitrogen (and to a lower extent to potassium, since it tends to evaporate at 700 °C rather than to stabilize), whose desorption was 63.8 % for BC500 and 46.1 % for BC700 at equilibrium, indicating the temperature-induced formation of aromatic heterocyclic rings converts nitrogen into a less soluble form [56]. As regards phosphorus, only a little difference in equilibrium values was observed (47.1 % and 48.4 % for BC500 and BC700, respectively), in contrast with desorption results on non-functionalized BC500 and BC700, where, at a phosphorus content of 4.6 and 1.1 mg/g, the observed C_e_ after 35 days were 21.9 and 3.9 mg/L, indicating desorption of 48 % and 35 % (for BC500 and BC700). However, the increase in phosphorous desorption could be due to the added phosphorus rather than the phosphorus naturally present in the matrix (which, on the contrary, showed reduced leachability with increasing temperature). This is further supported by an increase of the calculated K_des_, thus indicating BC700 may no longer exhibit the same affinity for phosphorus as BC500 because of its more alkaline pH_ZC_. Though biochar is known to be very affine to phosphate ions [69] the pH effect on potential ions desorption should be considered. Zheng et al. (2013) observed phosphorous desorption, in comparison to potassium and nitrogen, is more affected by pH, for pH values below 9 [52]. Therefore, the variation in the pH_ZC_ could contribute to the significant difference observed in K_des_ values. Yao et al. (2011) observed, that for pH values above 5, phosphate adsorption onto colloidal magnesium oxide (MgO) particles on the biochar surface (which is responsible for the affinity of phosphorus with biochar) is hindered [70]. Consequently, desorption is favored with increasing solution pH, which is governed by the pH_ZC_ (higher in BC700 compared to BC500).

As observed in other studies [71,72], the desorption kinetics were fitted by a second-order model (Equation (2)). The adequacy of the model is certified by the R^2^ values close to 1, as shown in Table 5 where the desorption rate constants, K_des_, for nutrients K, N, and P are reported; for the three investigated nutrients, the desorption kinetics in BC500 were characterized by lower desorption rates compared to BC700 [52].

**Table 5:** Kinetic study: nutrient desorption.

Nutrient source	BC500			BC700		
	K	N	P	K	N	P
**C**_**e**_**(mg·L**^**−**^**^1^)**	192	126	41	140	88	25
**K**_**des**_**(L·mg**^**−**^**^1^·days**^**−**^**^1^)**	0.0006	0.0008	0.0030	0.0017	0.0030	0.0128
**R**^**2**^	0.9742	0.9386	0.9829	0.9810	0.9845	0.9834

All the R^2^ values observed in BC700 were higher compared to BC500: this indicates that BC700 desorption behavior is better described by the model, likely due to the different pyrolysis conditions. The increase in pyrolysis temperature could decrease surface functional group activity, particularly those containing oxygen [73,74] which can interfere with nutrient desorption.

Additionally, it was observed the K_des_ was higher in the case of phosphorus, due to the diminished affinity between biochar and phosphate ions [75]. This reduced affinity can be due to electrostatic repulsion arising from the negatively charged surface of the biochar. Nitrate ions exhibited lower K_des_ values due to their reduced negative charge compared to phosphate. Furthermore, potassium displayed the lowest K_des_, indicating a stronger interaction with the biochar surface.

### 3.4 Effect of biochar on plantlet growth, leaf chlorophyll and carotenoid content

**Fig. 6:**
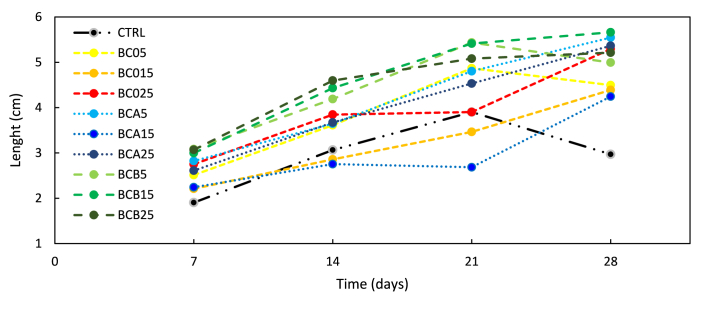
Plant height (cm) at 7, 14, 21, and 28 days of growth at different experimental conditions.

Tomato growth response to the applied biochar treatments at 7, 14, 21, and 28 days is shown in Fig. 6 and Table 6. The ANOVA analysis applied to the whole data set showed a statistically significant increase in plantlet height because of functionalized (BCA and BCB) and unfunctionalized (BC0) biochar application at days 21 and 28 of plant growth. Interestingly, the application of BCA at 5 % increased plant growth compared to BCA at 15 % while the application of BCB at 15 % increased plant growth compared to BCB at 5 %. This opposite trend on hypocotyl development could be explained as an effect due to the different ratios of macronutrients (N–P–K) in BCA and BCB respectively. N and K and their different ratios in BCB and BCA played a key role in different plant growth; in fact, the potassium content in the two biochars is different. Potassium regulates water content and hypocotyl expansion [76]. In seedlings grown in the presence of the lowest percentage of BCA used the potassium content was sufficient to enhance hypocotyl growth. Seedlings grown in the presence of BCB with a potassium content of about half that of BCA showed hypocotyl development comparable to BCA 5 % only at the percentages of BCB 15 and 25 %. At day 28, plants treated with unfunctionalized biochar (BC0) at 15 %, and 25 % were significantly higher than control plants, by 90 %, and 75 %, respectively. Tomato plants grown in soil supplemented with 25 % BCA and BCB were significantly higher than the control group (Fig. 5). BCA-treated plants were 78 % higher, while BCB-treated plants were 80 % higher compared to control plants. Furthermore, the treatment of plants with BCB at 5 % induced a significant increase in plant height compared to the control equal to 77 %. The increase of plant growth in response to the use of biochar as amendment was previously observed in *Phaseolus vulgaris* [77] and *Oryza sativa* [78], *Lactuca sativa* [79]*,* wheat, and bean species [80], and recently also in tomato plants, where biochar increased the growth of treated plants by 20 % compared to the control [81]. Unfunctionalized and functionalized biochar used in this study and obtained by the licorice industry could be proposed as a plant growth promoter to be utilized as a sustainable and effective amendment for tomato plant cultivation.

**Table 6:** Plant height (cm) at 7, 14, 21 and 28 days of growth at different experimental conditions. Means (±SD, n = 5) denoted by § indicates significant differences compared to control at the same day; * indicates significant differences compared to 15 % within the same group of treatment; p < *0.05*.

Treatments	Plant height (cm)			
	7 days	14 days	21 days	28 days
**Control**	1.9 ± 0.3	3.1 ± 0.4	3.9 ± 0.9	3.0 ± 0.3
**BC05**	3.1 ± 0.9	4.2 ± 1.0	5.4 ± 0.6*	5.0 ± 0.8
**BC015**	3.0 ± 0.8	4.4 ± 0.8	5.4 ± 0.8	5.7 ± 0.2§
**BC025**	3.1 ± 0.6	4.6 ± 1.2	5.1 ± 1.0	5.2 ± 2.0§
**BCA5**	2.5 ± 0.7	3.6 ± 1.3	4.9 ± 0.7*	4.5 ± 0.7
**BCA15**	2.2 ± 1.1	2.9 ± 1.5	2.8 ± 1.3	4.4 ± 0.7
**BCA25**	2.8 ± 0.4	3.8 ± 0.7	4.2 ± 0.7	5.3 ± 0.4§
**BCB5**	2.8 ± 0.7	3.6 ± 0.8	4.8 ± 0.8	5.3 ± 0.9§
**BCB15**	1.7 ± 1.2	2.1 ± 1.5	2.0 ± 1.5	4.0 ± 0.4
**BCB25**	2.0 ± 1.4	2.8 ± 2.0	3.4 ± 2.8	5.4 ± 1.9§

**Fig. 7:**
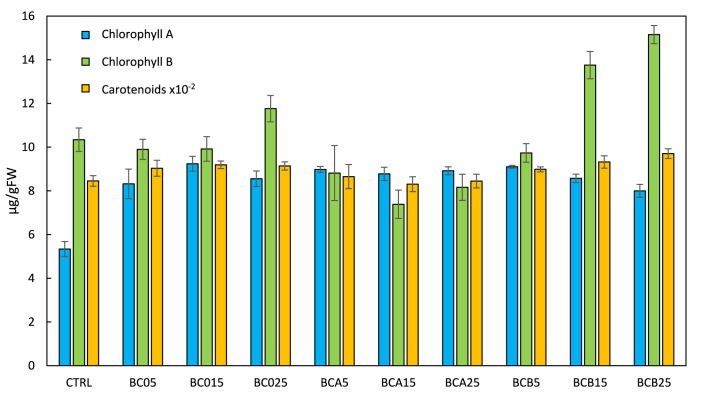
Leaf chlorophylls and carotenoids at 28 days of plant growth at different experimental conditions in micrograms on grams of fresh weight (μg/gFW).

Chlorophyll and carotenoid content analysis unveiled increased total chlorophyll (Fig. 7 and Table 7) in all biochar-treated plants, independently from the biochar type or percentage.

**Table 7:** Leaf chlorophylls and carotenoids at 28 days of plant growth at different experimental conditions. Means (±SD, n = 6) denoted by § indicates significant differences compared to control; * indicates significant differences compared to 5 % within the same group of treatment; ** indicates significant differences compared to 15 % within the same group of treatment; *p < 0.05.* *** indicates significant differences compared to 25 % within the different groups of treatment; *p < 0.05.*

Treatments	μg/gFW			
	Chlorophyll A	Chlorophyll B	Total chlorophyll	Carotenoids
**Control**	5.3 ± 0.3	10.3 ± 0.5	15.7 ± 0.7	845.4 ± 24.3
**BC05**	8.3 ± 0.7§	9.9 ± 0.5	18.2 ± 1.3§	903.7 ± 36.6
**BC015**	9.2 ± 0.3§*	9.9 ± 0.6	19.2 ± 0.2§	919.3 ± 17.2§
**BC025**	8.6 ± 0.4§	11.8 ± 0.6***	20.3 ± 0.3§*	914.0 ± 18.8
**BCA5**	9.0 ± 0.1§	8.8 ± 1.3	17.8 ± 1.4§	865.2 ± 55.2
**BCA15**	8.8 ± 0.3§	7.4 ± 0.6§	16.2 ± 0.8	830.5 ± 34.2
**BCA25**	8.9 ± 0.2§	8.2 ± 0.6§	17.1 ± 1.1**	844.6 ± 31.5
**BCB5**	9.1 ± 0.1§	9.7 ± 0.4	18.8 ± 0.5§	898.6 ± 11.4
**BCB15**	8.6 ± 0.2§	13.8 ± 0.6§*	22.3 ± 0.2§	932.2 ± 28.3§
**BCB25**	8.0 ± 0.3§*	15.2 ± 0.4§*	23.2 ± 0.1§***	970.4 ± 22.4§

As widely recognized, photosynthetic pigments play a pivotal role in influencing plant photosynthetic rates as indispensable factors for promoting healthy growth and productivity [82]. In this study, tomato seedlings treated with BC0 exhibited significantly higher chlorophyll *a* and *b* content compared to the control plants. Recent studies suggest biochar application to horticultural crops enhances photosynthetic pigments and rates under normal and stressed conditions. For instance, Jabborova et al. (2021) showed an increase (>35 %) in chlorophyll pigments in ginger plants (*Zingiber officinale*) treated with biochar compared to the control [83]. Likewise, biochar and humic acid treatments improved chlorophyll content in calendula (*Calendula officinalis* L.) compared to controls [84]. Similar results were by Simiele et al. (2022) in tomato plants treated with biochar obtained from cotton sticks and orchard pruning biomass [85]. Almorai et al. (2020) observed, that amendmenting the soil with biochar (10 ton·ha^−1^) increased the amount of chlorophyll *a*, *b*, and carotenoids by 51, 9, and 35 % above the control in *Solanum lycopersicum* [86] in line with this study (the increase was respectively by 50, 47 and 15 % above the control). The application of biochar showed positive effects on plant fitness in the early seed development phase of *Solanum lycopersicum*. The results obtained suggest that enriching the soil with this biochar enables the plant to obtain an additional source of the nutrients necessary for growth. However, different effects of increased plant fitness can be appreciated depending on the percentage and type of biochar tested. Nitrogen (N) is an element with a key role in chlorophyll production and protein synthesis, in fact when nitrogen is deficient plants develop yellow leaves and their growth is stunted [76]. The use of biochar as a soil conditioner has improved the bioavailability of this element and increased the hypocotyl height and chlorophyll content in leaves compared to the control. The highest amount of total chlorophyll was measured in plants treated with 25 % functionalized BCB. This result shows that the release of nitrogen by the biochar increases the chlorophyll content in the plant allowing for better fitness [76]. The highest total chlorophyll amount in plants treated with functionalized BCB at 25 % was observed. This trend also in chlorophyll *a* and *b* and carotenoid content was observed (Fig. 6). Plants treated with BCB at 15 % and 25 % and BC0 at 15 % showed significantly higher carotenoids than control plants. Similar results were reported by Shamim et al. (2015), demonstrating the presence of biochar and N–P–K fertilizer products improved plant fitness and photosynthetic capacity [87]. The N element in NPK fertilizer has the function of preparing amino acids (proteins), nucleic acids, nucleotides, and chlorophyll in plants. P has a function as a storage and energy transfer, and it is involved in the photosynthesis process. Finally, the K acts as an enzyme activator, and assists in the transport of assimilated results from the leaf to the plant tissue, improving plant health, growth, and nutrition [88]. In this study, the ratio 4:1:3 (BCB biochar) resulted in the optimal ratio to improve plant growth and fitness. On the other hand, the addition of macronutrient-rich biochar with a consistent N content could minimize soil N loss by NH_3_ volatilization and the denitrification of N_2_ and N_2_O, increasing soil organic matter and soil cation exchange capacity. Macronutrients are essential for plant growth and development, but it is crucial to consider the stoichiometric ratios with which these nutrients are administered to avoid potential negative effects or mitigate potential adverse effects.

## 4 Conclusions

Biochar (BC500 and BC700) was synthesized and used as an effective *Solanum lycopersicum* soil conditioner by enhancing licorice processing waste.

The carbonization temperature (500 and 700 °C) significantly varied the properties of biochar; the yield of biochar production decreased with increasing temperature. Volatilization of phosphorus and potassium and less pronounced volatilization of nitrogen occurred at higher temperature, resulting in biochar depletion from 10 to 8 mg/g for K, 5 to 1 mg/g for P, and 5 to 4 mg/g for N. The solubility of nutrients decreased with increasing temperature due to their conversion to less water-soluble materials.

Specific surface area (BET), due to the increase in pore size (from 8 to 30 nm) and decrease in particle size, increased with increasing temperature from 13 to 21 m^2^/g pH_ZC_ increased with increasing temperature from 7.5 to 8.4.

Analyses carried out on nutrient desorption kinetics showed functionalized biochar was an excellent slow-release nutrient carrier material, and desorption kinetics can be modulated by pyrolysis temperature. Specifically, biochar produced at 500 °C desorbed nutrients at a slower rate than that produced at 700 °C. Equilibrium was reached after 14 days compared with 10 days for BC700. In addition, the K_des_ of BC500 were lower than BC700 for all 3 NPK nutrients. The application of unfunctionalized and functionalized biochar (BC500) as a soil conditioner significantly increased tomato plant length and chlorophyll and carotenoid content compared with the control. Notably, the observed parameters depended heavily on both biochar content and NPK ratio used showing that optimization of these parameters is appropriate to maximize crop production. The best treatment, employing functionalized biochar with NPK ratio 4:1:3 at 25 % relative to the soil, produced a 38 % increase in chlorophyll amount and a 15 % increase in carotenoid content. Biochar also proved to be an engineerable material by intervening on the carbonization temperature.

**Table:** Notations

**Symbol**	**Description**
BC500	Biochar produced at 500 °C
BC700	Biochar produced at 700 °C
NPK	Nitrogen, phosphorus and potassium ratio in weight
F.BC500	Biochar produced at 500 °C and functionalized
F.BC700	Biochar produced at 700 °C and functionalized
CTRL	Control: biochar-free samples
BC0X	Unfunctionalized biochar produced at 500 °C where X is biochar % in soil
BCAX	Functionalized biochar produced at 500 °C with NPK ratio 3:1:4
BCBX	Functionalized biochar produced at 500 °C with NPK ratio 4:1:3

## Resource availability

### Lead contact

Further information and requests for resources and reagents should be directed to and will be fulfilled by the lead contact, Luca Di Palma (luca.dipalma@uniroma1.it).

## Data availability

All data reported in this paper will be shared by the lead contact upon request.

Any additional information required to reanalyze the data reported in this paper is available from the lead contact upon request.

## CRediT authorship contribution statement

**Domenico Rosa:** Writing – original draft, Investigation, Formal analysis, Conceptualization. **Valerio Petruccelli:** Writing – original draft, Investigation, Data curation, Conceptualization. **Maria Cristina Iacobbi:** Investigation. **Elisa Brasili:** Validation, Methodology, Formal analysis, Data curation. **Camilla Badiali:** Validation, Methodology, Investigation, Formal analysis, Data curation. **Gabriella Pasqua:** Writing – review & editing, Validation, Supervision, Conceptualization. **Luca Di Palma:** Writing – review & editing, Validation, Supervision, Conceptualization.

## Declaration of competing interest

The authors declare that they have no known competing financial interests or personal relationships that could have appeared to influence the work reported in this paper.
